# LRP-1 functionalized polymersomes enhance the efficacy of carnosine in experimental stroke

**DOI:** 10.1038/s41598-020-57685-5

**Published:** 2020-01-20

**Authors:** Eun-Sun Kim, Donghyun Kim, Sophie Nyberg, Alessandro Poma, Denis Cecchin, Saurabh A. Jain, Kyeong-A Kim, Young-Jun Shin, Eun-Hye Kim, Minyeong Kim, Seung-Hoon Baek, Jin-Ki Kim, Thorsten R. Doeppner, Ali Ali, Jessica Redgrave, Giuseppe Battaglia, Arshad Majid, Ok-Nam Bae

**Affiliations:** 1 College of Pharmacy Institute of Pharmaceutical Science and Technology, Hanyang University, Ansan, 15588 Republic of Korea; 2 Department of Chemistry, London, England; 3 Institute of Physics of Living System, University College London, London, WC2N 5DU England; 4 Sheffield Institute for Translational Neuroscience, University of Sheffield, Sheffield, S10 2TN England; 5 College of Pharmacy and Research Institute of Pharmaceutical Science and Technology (RIPST), Ajou University, Suwon, KS002 Republic of Korea; 6 Department of Neurology, University Medical Center Goettingen, Goettingen, 37075 Germany

**Keywords:** Molecular neuroscience, Stroke

## Abstract

Stroke is one of the commonest causes of death with limited treatment options. L-Carnosine has shown great promise as a neuroprotective agent in experimental stroke, but translation to the clinic is impeded by the large doses needed. We developed and evaluated the therapeutic potential of a novel delivery vehicle which encapsulated carnosine in lipoprotein receptor related protein-1 (LRP-1)-targeted functionalized polymersomes in experimental ischemic stroke. We found that following ischemic stroke, polymersomes encapsulating carnosine exhibited remarkable neuroprotective effects with a dose of carnosine 3 orders of magnitude lower than free carnosine. The LRP-1-targeted functionalization was essential for delivery of carnosine to the brain, as non-targeted carnosine polymersomes did not exhibit neuroprotection. Using Cy3 fluorescence *in vivo* imaging, we showed that unlike non-targeted carnosine polymersomes, LRP-1-targeted carriers accumulated in brain in a time dependent manner. Our findings suggest that these novel carriers have the ability to deliver neuroprotective cargo effectively to the brain.

## Introduction

Stroke is a serious, life-threatening medical condition that occurs when the blood supply to a part of the brain is restricted. Stroke is the fifth most frequent cause of death globally and a leading cause of serious long-term disability^1,2^. There has been a significant worldwide increase in stroke burden over the last two and half decades. There has been a significant increase in stroke burden in the world over the last two and half decades. Stroke treatment in the acute phase generally includes medicines to lyse and/or remove blood clots. Currently, the only approved drug for acute stroke is recombinant tissue plasminogen activator (rtPA, alteplase), which increases the risk for subsequent hemorrhage and has a limited time window for administration. As a consequence, only a small percentage of patients receive rtPA treatment. There are no approved treatments for the numerous damaging pathological processes that are activated during stroke. These include excitotoxicity, oxidative stress, apoptosis, edema, inflammation, and impairment of the blood-brain barrier (BBB). The development of a neuroprotective treatment that has the ability to favorably influence the multiple deleterious mechanisms that are activated during stroke is an urgent clinical need. Moreover, combining neuroprotection with reperfusion therapy has been proposed as a strategy in the development of acute stroke therapies^3^.

Carnosine is an endogenous pleiotropic dipeptide consisting of alanine and histidine that is expressed in many tissues of the body, including the central nervous system (CNS)^4–6^. Recent studies have shown that carnosine has beneficial effects against various diseases including brain-related disorders, and that carnosine shows robust neuroprotective effects in acute ischemic stroke^4,7–10^. It exhibits many properties that make it an attractive neuroprotective agent including antioxidant, anti-excitotoxic, anti-matrix metalloproteinase, and metal ion chelating and intracellular pH buffering properties^7,11,12^. Despite its potent biological benefit against ischemic stroke in animal models, clinical translation of this compound has been impeded by the large doses needed (500-1,000 mg/kg) for efficacy^6,13^. High doses may be required possibly due to the rapid breakdown of carnosine after systemic administration due to the action of serum and cellular proteases^14^. The use of carriers that are able to prevent serum degradation of carnosine, as well as target delivery to the CNS may significantly reduce the doses needed and therefore could help in translating the promising preclinical findings into clinical studies.

Polymersomes are synthetic vesicles formed by the self-assembly of amphiphilic co-polymers in aquatic conditions^15^. In this study we have used pH-sensitive poly(2-(diisopropylamino)ethyl methacrylate) (PDPA) based polymersomes, poly[oligo(ethylene glycol) methyl methacrylate] (POEGMA) which have been functionalized by incorporating Angiopep-2 on to their surface, targeting lipoprotein receptor related protein-1 (LRP-1) which is one of the highly expressed receptors in the BBB^16–18^. We have previously shown that these POEGMA-PDPA polymersomes functionalized with Angiopep-2, enables non-invasive entry of polymersomes across the BBB by transcytosis and the delivery of drugs and macromolecular cargo such as antibodies to the cellular cytoplasm of CNS cells^15,19^.

In this study, we assessed the neuroprotective potential of carnosine encapsulated in polymersomes with Angiopep-2 for LRP-1-mediated targeted delivery to the CNS in experimental stroke. We hypothesized that the delivery of carnosine encapsulated within Angiopep-2-POEGMA-PDPA would be able achieve the same therapeutic effects observed with free carnosine at a much lower dosage, due to the targeting peptide and due to encapsulation of carnosine protecting it from premature degradation. We also evaluated the time dependent distribution of targeted and non-targeted carnosine encapsulated polymersomes in brain and other tissues.

## Results

### Characterization of brain-targeted polymersomes loaded with carnosine

**Figure 1:**
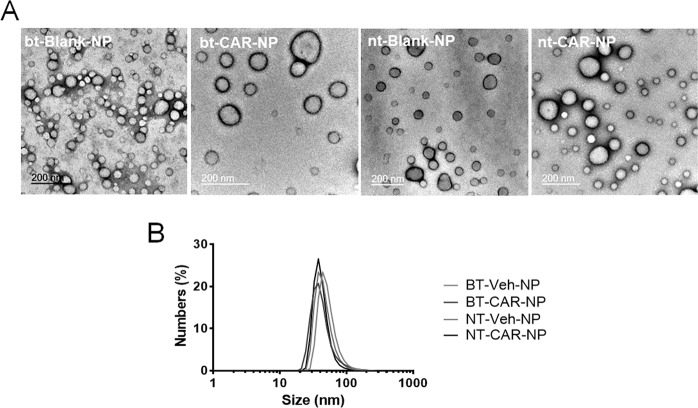
Morphology and size distribution of polymersomes (**A**) Polymersome morphology and size were characterized via transmission electron microscopy. Electron micrographs confirm the vesicular structure of all polymersome types; bt-Blank-NP, bt-CAR-NP, nt-Blank-NP and nt-CAR-NP, with a distribution of diameters within each sample. (**B**) Polymersome size was assessed by dynamic light scattering (DLS). Size distributions of polymersomes were similar across all populations, with an average diameter of around 40 nm.

The formation of POEGMA–PDPA polymersomes were carried out by the hydration of a polymer film at pH 7.4. Transmission electron microscopy (TEM) confirmed that POEGMA–PDPA copolymer alone or in combination with carnosine formed vesicles from the film hydration method, resulting in a distribution of nano-polymersome sizes (Fig. 1A). Polymersome size, and size preservation after loading of the drug, was quantified by measuring the polymersome size by dynamic light scattering (DLS). DLS data analysis by the number of vesicles of any given diameter per sample demonstrated that the most abundant sizes were similar across all samples (approx. 40–60 nm), for both blank (Blank-NPs) and carnosine-loaded polymersomes (CAR-NPs) (Fig. 1B) regardless of Angiopep-2 incorporation (Brain-targeted (bt-) or Non-targeted (nt-)).

### Improvement of histological and functional outcomes by brain-targeted polymersomes loaded with carnosine in rats with permanent focal ischemia

**Figure 2:**
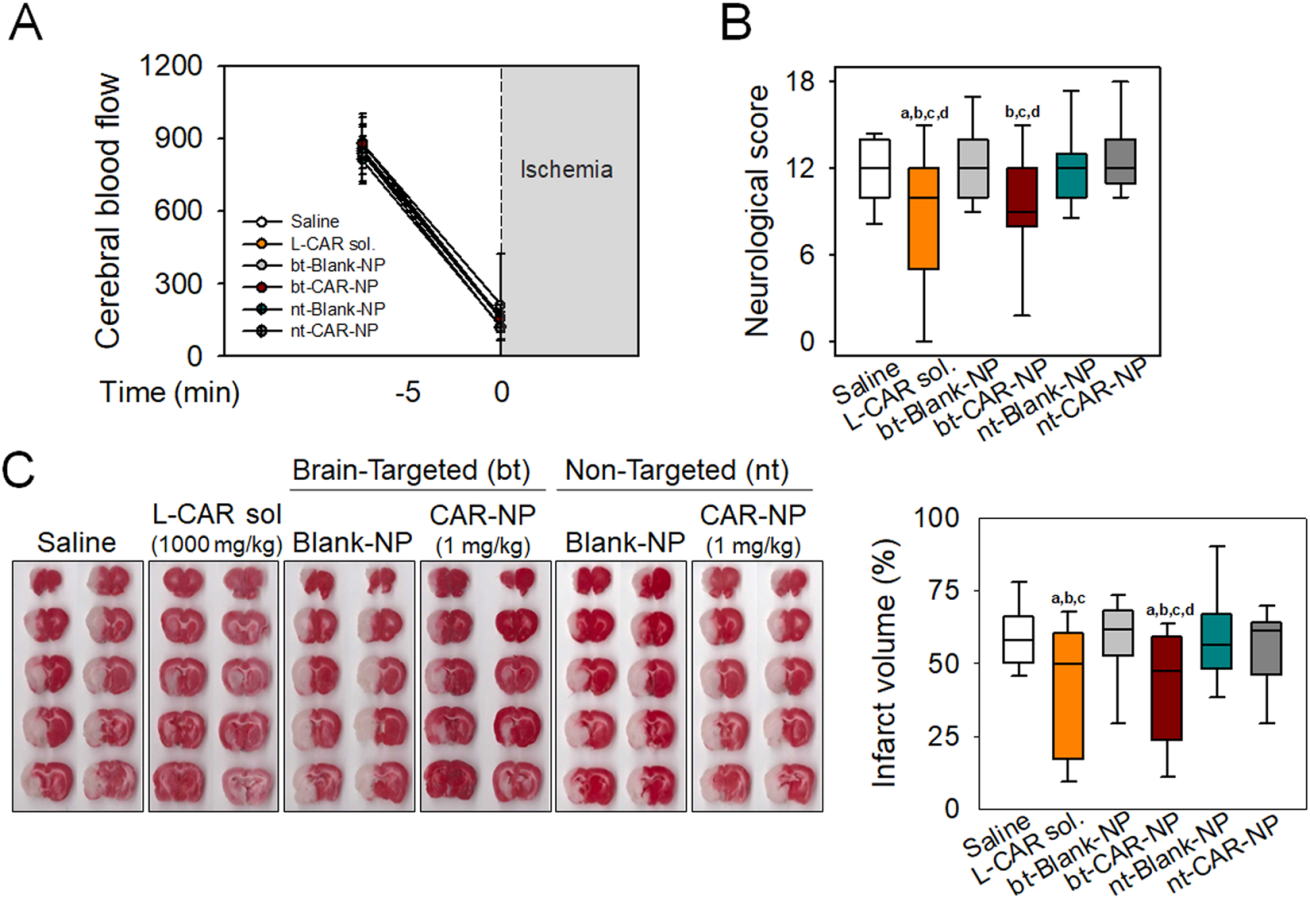
Protective effects of brain-targeted polymersomes with carnosine on neurological function and infarct volume in rats with pMCAo Permanent focal ischemia was induced in rats by middle cerebral artery occlusion (MCAo) using intraluminal suture. Carnosine (1,000 mg/kg), bt-CAR-NP (1 mg/kg), nt-CAR-NP (1 mg/kg) or corresponding vehicles (Saline, bt-Blank-NP and nt-Blank-NP, respectively) were intravenously administered at 3 hours after ischemic onset. (**A**) Cerebral blood flow was monitored before and at the onset of pMCAo. Values are mean ± SD. (**B**) Functional deficits were examined with neurological severity scores at 24 hours after ischemia. (**C**) Infarct volume was calculated using 2,3,5-triphenyltetrazolium chloride (TTC) staining of brain sections. The infarct volume for each section was determined and edema correction was performed by measurement of the infarcted and control hemisphere. Representative scanned images were shown. Box plot: median, first and third quartile; whiskers: range. All values were analyzed by one-way ANOVA. Different letters indicate a statistically significant difference (p < 0.05) between experimental groups: a vs. rats treated with saline; b vs. rats treated with bt-Blank-NP; c vs. rats treated with nt-Blank-NP; d vs. rats treated with nt-CAR-NP. A to C: N = 15 rats/group.

To determine the efficacy of polymersomes loaded with carnosine, permanent focal ischemia was induced in rats by occlusion of middle cerebral artery (MCAo) using an intraluminal monofilament. No significant differences among the experimental groups were detected in physiological variables of body weight and rectal temperature (data not shown). Changes in cerebral blood flow (CBF) before and after ischemia were not significant between the groups (Fig. 2A).

#### Functional outcomes

Treatment with carnosine (L-CAR sol.; 1,000 mg/kg) at 3 hours after ischemia onset significantly reduced neurological deficits induced by permanent MCAo. Remarkably, bt-CAR-NP exhibited similar reduction in neurological deficits at a concentration of 1 mg/kg (Fig. 2B). On the other hand, nt-CAR-NP (1 mg/kg) did not alter functional outcomes.

#### Histological outcomes

Post-treatment with carnosine (1,000 mg/kg) or bt-CAR-NP (1 mg/kg) at 3 hours after ischemia significantly reduced cerebral infarct volumes in rats following permanent MCAo. Infarct volumes expressed as a percentage of the ischemic hemisphere were 59.6 ± 11.2% and 57.6 ± 16.7% in saline and bt-Blank-NP-treated rats respectively (mean ± SD). Rats treated with carnosine (1,000 mg/kg) or bt-CAR-NP (1 mg/kg) had significantly smaller infarct volumes of 43.1 ± 22.0% and 42.5 ± 19.0%, respectively (mean ± SD).

### Protective effects of brain-targeted polymersomes loaded with carnosine against brain damage induced by ischemia/reperfusion

**Figure 3:**
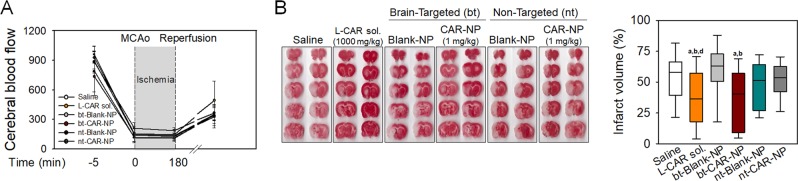
Protective effects of brain-targeted polymersomes with carnosine on infarct volume in rats with tMCAo Transient focal ischemia was induced in rats for ischemia (3 hours)/reperfusion (21 hr) injury (I/R injury), and brain damage was calculated with histological analysis. Carnosine (1,000 mg/kg), bt-CAR-NP (1 mg/kg), nt-CAR-NP (1 mg/kg) or corresponding vehicles (Saline, bt-Blank-NP and nt-Blank-NP, respectively) were intravenously administered at 3 hours after ischemic onset. (**A**) Cerebral blood flow before and after I/R injury. Values are mean ± SD. (**B**) Infarct volume was calculated using TTC staining of brain sections. Representative scanned images were shown. Box plot: median, first and third quartile; whiskers: range. All values were analyzed by one-way ANOVA. Different letters indicate a statistically significant difference (p < 0.05) between experimental groups: a vs. rats treated with saline; b vs. rats treated with bt-Blank-NP; d vs. rats treated with nt-CAR-NP. A and B: N = 15 rats/group.

To determine if bt-CAR-NP reduces brain damage induced by ischemia/reperfusion (I/R), a transient rat MCAo model was used. Changes in CBF confirmed I/R in the brain (Fig. 3A), which were not significantly different between the treatment groups. At 3 hours after ischemia, carnosine (1,000 mg/kg), bt-CAR-NP (1 mg/kg), nt-CAR-NP (1 mg/kg) or corresponding vehicle were intravenously administered to rats, and reperfusion was initiated. While nt-CAR-NP (1 mg/kg) was not effective in reducing infarct volumes (46.3 ± 19.7% in nt-Blank-NP-treated rats vs. 51.8 ± 14.2% in nt-CAR-NP-treated rats, mean ± SD), carnosine (1,000 mg/kg) or bt-CAR-nt (1 mg/kg) significantly reduced brain infarct volumes after I/R injury (Fig. 3B) (53.4 ± 19.7% in saline-treated rats vs. 36.5 ± 24.1% in carnosine-treated rats; 59.3 ± 22.0% in bt-Blank-NP-treated rats vs. 36.9 ± 22.7% bt-CAR-NP-treated rats, mean ± SD).

**Figure 4:**
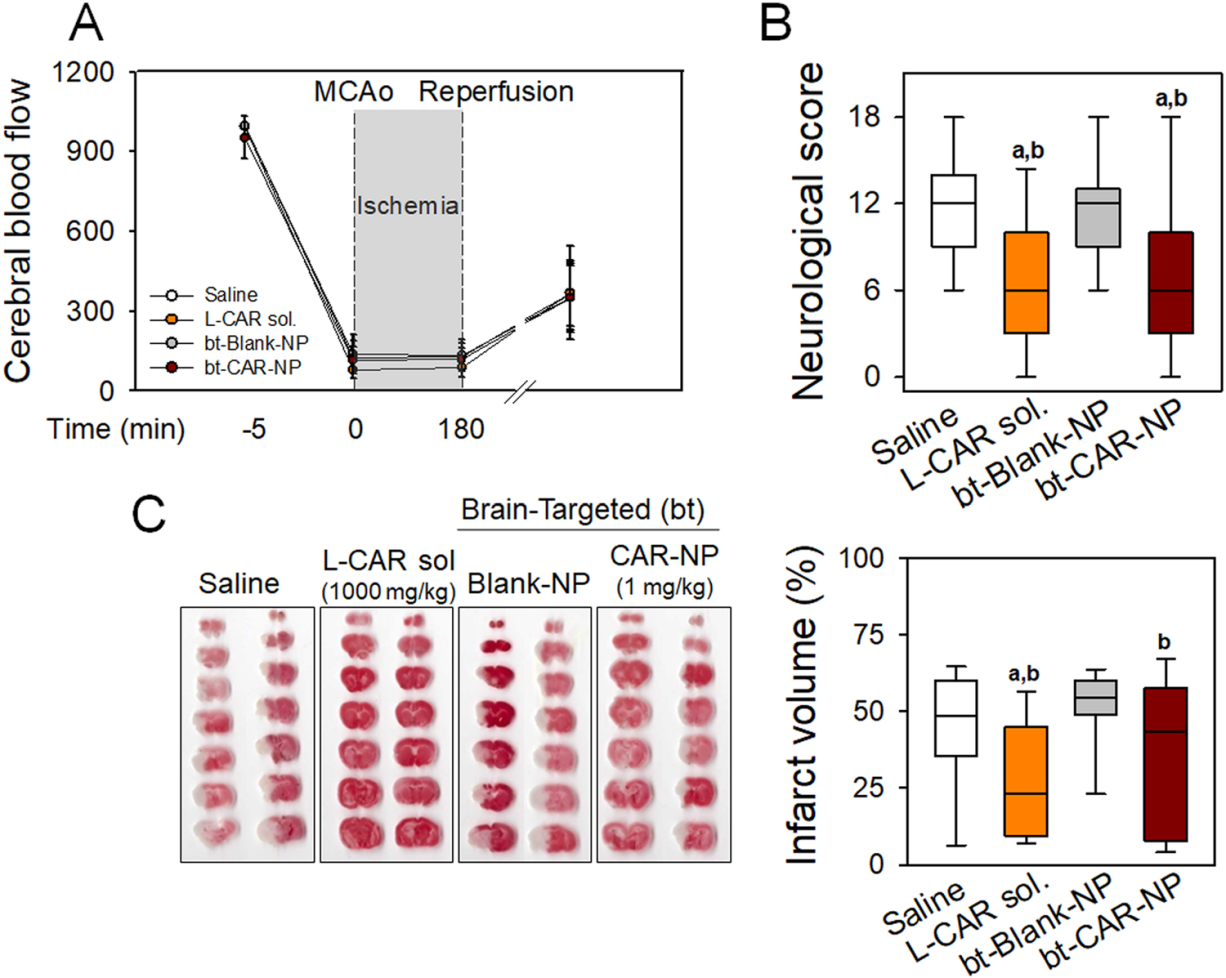
Improvement of functional and histological damage by brain-targeted polymersomes with carnosine in mice with tMCAo Transient focal ischemia (3 hour ischemia/21 hour reperfusion) using intraluminal suture was induced in mice. Carnosine (1,000 mg/kg), bt-CAR-NP (1 mg/kg), or corresponding vehicles (Saline and bt-Blank-NP, respectively) were intravenously administered at 3 hours after ischemic onset. (**A**) Cerebral blood flow was monitored before and after I/R injury in mice. Values are mean ± SD. (**B**) Functional deficits were examined with neurological severity scores at 24 hours after ischemic onset. (**C**) Infarct volume was calculated using TTC staining of brain sections. Representative scanned images were shown. Box plot: median, first and third quartile; whiskers: range. All values are analyzed by one-way ANOVA. Different letter indicates a statistically significant difference (p < 0.05) between experimental groups: a vs. mice treated with saline; b vs. mice treated with bt-Blank-NP. A to C: N = 15 mice/group.

To determine whether the protective effect of bt-CAR-NP is species-specific, carnosine or bt-CAR-NP were administered to mice with transient focal ischemia. Changes in CBF were monitored to confirm I/R injury (Fig. 4A). Improvement of functional behavioral deficits and histological brain damage were observed in mice treated with carnosine (1,000 mg/kg) or bt-CAR-NP (1 mg/kg) (Fig. 4B,C) (43.4 ± 21.1% in saline-treated mice vs. 28.1 ± 19.3% in carnosine-treated mice; 50.7 ± 14.3% in bt-Blank-NP-treated mice vs. 35.2 ± 25.6% bt-CAR-NP-treated mice, mean ± SD).

### *In vivo* fluorescence imaging for tissue distribution of polymersomes

**Figure 5:**
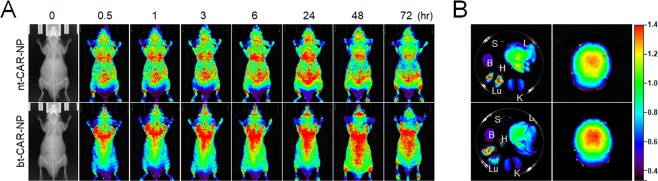
*In vivo* fluorescent imaging of mice after intravenous injection with polymersomes with carnosine Cy3-labeled brain-targeted or non-targeted polymersomes were intravenously injected in BALB/c mice. (**A**) Fluorescence signal captured by IVIS Lumina XR Imaging System in whole mice at 0, 0.5, 1, 3, 6, 24, 48 and 72 hours after injection with bt-CAR-NP (1 mg/kg) or nt-CAR-NP (1 mg/kg). (**B**) *Ex vivo* imaging of isolated organs including spleen (S), liver (L), brain (B), heart (H), kidney (K), and lung (Lu) at 24 hours after injection with polymersomes. Representative images are shown. A and B: N = 4.

Next, we examined tissue distribution of the polymersomes using Cy3-fluorescence longitudinally over time. Longitudinal *in vivo* imaging showed that functionalized polymersomes targeting the LRP-1 receptor resulted in accumulation of Cy3 fluorescence in the brain. Compared with nt-CAR-NP, the fluorescence signal of bt-CAR-NP in the brain increased gradually from 3 hours to 48 hours, and decreased at 72 hours after injection (Fig. 5A). In *ex vivo* fluorescence imaging of organs (liver, lung, spleen, heart, kidney, and brain) isolated at 24 hours after injection, the fluorescent signal from bt-CAR-NP in brain showed higher intensity than that from nt-CAR-NP (Fig. 5B).

## Discussion

Rapid delivery of systemically administered neuroprotective agents across the BBB into the ischemic brain region is a critical component of stroke neurotherapeutic strategies^20^. One of the reasons for the lack of efficacy of previously tested neuroprotective agents is the lack of penetration of the agent across the BBB^21^. Nanomaterial-based therapeutics or imaging agents have been approved by US FDA, and interest in these nanomedicines is now grwoing^22^. Recent advances in nanomaterials enable the targeted delivery of therapeutic agents to a specific organ such as brain. Nanoparticles may enhance delivery of neuroprotective drugs that in their free form cannot pass the BBB, or do so in very low amounts and thereby not achieving therapeutic concentrations^23–25^.

Here, we have used pH-sensitive POEGMA –PDPA polymersomes which have been functionalized by incorporating LRP-1-targeted Angiopep-2 on to their surface. These polymersomes have the ability to enter cells via receptor-mediated endocytosis, and are then trafficked to endosomes where the reduction in pH in the microenvironment triggers polymersome dissociation, conformational change and as a consequence, the polymersomes disintegrate to produce many individual copolymer chains. This triggers an elevation in the osmotic pressure that temporarily lyses the endosomal membrane, allowing the release of encapsulated drug into the cell cytosol^26–28^.

Using *in vivo* and *ex vivo* imaging, (Fig. 5) we tested the ability of LRP-1-targeted (with Angiopep-2) versus non-targeted POEGMA-PDPA polymerases to cross the BBB (bt-CAR-NP vs. nt-CAR-NP), Cy3-labeled polymersomes after administration in the tail vein of nude mice. Our findings showed strong fluorescence of Cy3 in the brain for up to 48 hours in the group treated with LRP-1-targeted polymersomes. On the other hand, non-targeted polymersomes were uniformly distributed throughout the body, but the degree of Cy3 fluorescence distributed to the brain was relatively low compared to the LRP-1-targeted polymersomes. This suggests that the LRP-1-targeted polymersomes had enhanced delivery to the brain. These data show, for the first time, that targeted polymersomes have the potential to cross the BBB, penetrate brain tissue, and deliver neuroprotective cargo. Carnosine encapsulation in LRP-1-targeted polymersomes reduces the dose requirement of carnosine for efficacy by at least 3 orders of magnitude. (1 mg/kg intravenous carnosine encapsulated in LRP-1-targeted polymersomes exhibited similar efficacy to 1,000 mg/kg intravenous dosage of carnosine). We have also shown that merely encapsulating carnosine in polymersomes without targeting is insufficient for efficacy at the doses tested. LRP-1-targeting, which induces transcytosis of the polymersomes across the BBB, is essential for efficacy and entry of carnosine into the brain.

There are some limitations of our study. First, we did not test the effect of smaller or larger doses of encapsulated carnosine. It is possible that even smaller doses of carnosine encapsulated in LRP-1-targeted polymersomes is efficacious. Similarly, it is possible that non-targeted polymersomes containing doses of carnosine higher than 1 mg/kg would have been efficacious. Future studies will also need to determine the serum and brain kinetics of encapsulated carnosine. To detect *in vivo* fluorescence, nude mice were used to minimize interfering or blocking of light by animal hair in this study. Further kinetic studies may need to be done in normal mice to exclude any potential effect of immunodeficiency on behavior of carnosine-encapsulated nanopolymersomes. Second, different treatment regimens and treatment at different time points after onset of ischemia would be interesting and useful for therapy development. Third, while this study was designed to demonstrate the proof of concept of efficacy of LRP-1-targeted carnosine encapsulation, additional studies in females, older animals, and animals with co-morbidities are still needed to fully satisfy the Stroke Therapeutic Academic Industry Roundtable (STAIR) recommendations^29^.

In summary, our data show that carnosine encapsulated in LRP-1-targeted polymersomes is effective in reducing cerebral injury and improving neurological function after permanent and transient ischemic stroke. We also show that that this novel delivery vehicle for carnosine requires the administration of much lower doses of carnosine, which facilitates translation of these preclinical findings to clinical studies. Moreover, our findings open up the possibility that other promising neuroprotective agents could be encapsulated in these novel delivery vehicles for targeted CNS delivery.

## Methods

2-(Methacryloyloxy)ethyl phosphorylcholine (MPC; >99% purity) was donated by Biocompatibles UK Ltd (Farnham, UK). 2-(Diisopropylamino)ethyl methacrylate (DPA) was purchased from Scientific Polymer Products (Ontario, US). Carnosine, poly(ethylene glycol) methyl ether methacrylate [P(OEG_10_MA)], copper (I) bromide, 2,2′-bipyridine, methanol, bovine liver catalase, glucose oxidase, glucose, 2,3,5-triphenyltetrazolium chloride, paraformaldehyde, isopropanol and the gel filtration column for the purification of the polymersomes was made with Sepharose 4B were purchased from Sigma-Aldrich (St. Louis, MO, USA). The silica used for the removal of the atom transfer radical polymerization copper catalyst was column chromatography grade silica gel 60 (0.063 to 0.200 mm) purchased from Merck (Darmstadt, Germany). 2-(N-morpholino) ethyl 2-bromo- 2-methylpropanoate initiator was synthesized according to a previously report^30^. The polymersomes were labeled using Cy3 purchased from Lumiprobe (Hunt Valley, MD, USA). PBS was made from Oxoid tablets. Silicone-coated 4-0 and 6-0 monofilament nylon sutures were purchased from Doccol Co. (Sharon, MA, USA). Isoflurane was obtained from Hana Pharm (Hwaseong, Korea).

### Polymersome preparation and encapsulation of carnosine

Cy3-labeled polymersomes were prepared from the POEGMA-PDPA polymer or Angiopep-2-POEGMA-PDPA, previously prepared and characterized as previously described^15^. Four types of nanopolymersomes (Angiopep-2-POEGMA-PDPA vehicle (Brain-targeted blank nanopolymersomes; bt-Blank-NP), Angiopep-2-POEGMA-PDPA with carnosine (Brain-targeted carnosine-loaded nanopolymersomes; bt-CAR-NP), POEGMA-PDPA blank (Non-targeted vehicle nanopolymersomes; nt-Blank-NP), and POEGMA-PDPA with carnosine (Non-targeted carnosine-loaded nanopolymersomes; nt-CAR-NP).

For encapsulation of cargo into polymersomes (bt-CAR-NP and nt-CAR-NP), electroporation was performed as previously reported^31^. A stock solution of carnosine was filter sterilized and loaded together with polymersomes into 800 μL electroporation cuvettes (BioRad; Hercules, CA, USA), to a final concentration of 1.5 mg/mL carnosine and 3 mg/mL polymersomes, and electroporation was performed with an Eppendorf 2510 electroporator (Hamburg, Germany) with a total of 10 pulses at 2500 V. Electroporated polymersomes were left in 4 °C overnight to allow sufficient time for the pores to close, followed by purification via gel permeation chromatography to remove residual free protein, with Sepharose 4B as the mobile phase. Purified polymersomes were assessed for size and shape with dynamic light scattering (DLS) and transmission electron microscopy (TEM), respectively. Protein encapsulation was determined via reverse-phase high-performance liquid chromatography (RP-HPLC) and drug encapsulation efficiency in polymersomes was calculated with a MATLAB script utilizing DLS data to determine the average number of drug molecules per polymersome.

### Quantification of polymersome cargo content with HPLC

High Performance Liquid Chromatography (HPLC) was performed using a Dionex Ultimate 3000 instrument (Thermo Fisher Scientific; Rockford, IL, USA). To detect total polymer and protein content, polymersomes were disrupted in 0.05% trifluoroacetic acid (TFA) (Sigma-Aldrich). A multi-step gradient of HPLC grade methanol (Sigma-Aldrich) as eluent A and milliQ water (Merck Millipore; Burlington, MA, USA) in 0.05% trifluoroacetic acid as eluent B was used to elute samples over 30 minutes with a C18 column (Jupiter C18 300 Å, 150 × 4.60 mm, 5 μm; Phenomenex®; Torrance, CA,USA) as reported previously, with a mobile phase gradient of 95% mQH2O + 0.05% TFA and 5% MeOH + 0.05% TFA and a flow rate of 1 mL/min^21^. Polymer content was detected using fluorescence (λex: 540 nm; λem: 560 nm). Protein content was detected using the UV/Vis channel of 220 nm. Areas under the detected peaks were measured using Chromeleon software (Thermo Fisher Scientific).

### Dynamic light scattering

The sample was analyzed with a 120-mW He-Ne laser at 630 nm at a controlled temperature of 25 °C, and the scattered light was measured at an angle of 173°. For the analysis, the sample was diluted with filtered PBS (pH 7) at a final concentration of 0.2 mg/mL into a final volume of 500 μL and then transferred into a polystyrene cuvette (DTS0012; Malvern Instruments; Malvern, UK). All DLS data were processed using Dispersion Technology Software (Malvern Instruments).

### Transmission electron microscopy

A phosphotungstic acid (PTA) solution was used as a positive and a negative staining agent because of its preferential interaction with the ester groups on the poly-MPC polymers. The PTA staining solution was prepared by dissolving 37.5 mg of PTA in boiling distilled water (5 mL). The pH was adjusted to 7.4 by adding a few drops of 5 M NaOH with continuous stirring. The PTA solution was then filtered through a 0.2 µm filter. 5 µL of polymersome/PBS dispersion was deposited onto glow-discharged copper grids. After 1 min, the grids were blotted with filter paper and then immersed into the PTA staining solution for 5 s for positive staining and 10 s for negative staining. Then, the grids were blotted again and dried under vacuum for 1 min. Grids were imaged using an FEI Tecnai G2 Spirit TEM microscope at 80 kV (FEI Company, Hillsbro, OR, USA).

### Animals

Experiments and procedures were performed in accordance with the institutional and international guidelines and regulations. All protocols for animal experiments were approved by the Institutional Animal Care and Use Committee (IACUC; 2016-0108A and 2016–0219A) at Hanyang University, Korea. Five-week-old male Sprague-Dawley rats (230–300 g), six-week-old male C57BL/6 mice (20–25 g) or male BALB/c nude mice (20–25 g) were purchased from Koatech (Pyeongtaek, Korea). Animals were housed in a specific-pathogen-free zone (12 h light/dark cycle, 23 °C, and 50% humidity) and randomly divided into the treatment groups. Investigators were blind to the allocation of treatment during surgeries and outcome evaluations. A total of one hundred and eighty animals (Fifteen rats in each group; six groups; two models of permanent and transient ischemia) were used to determine the efficacy of carnosine or carnosine-loaded nanopolymersomes in rat models of ischemia. Sixty C57BL/6 mice were used for mouse models of transient ischemia, and eight BALB/c nude mice were used to conduct *in vivo* imaging of nanopolymersomes.

### Ischemic stroke model in rats and mice

Permanent or transient brain ischemia was induced by middle cerebral artery occlusion (MCAo)^13^. Anesthesia was induced by isoflurane inhalation and maintained during the surgical procedure. Rectal temperature was monitored and maintained at 37 °C during surgery. The measurement of cerebral blood flow (CBF) was performed by laser Doppler (Perimed; North Royalton, OH, USA) before and at the onset of MCAo, and after reperfusion. The success of MCAo was confirmed by a drop in the regional CBF in the left cerebral cortex to less than 30% of pre-MCAo values^32^. The left common carotid artery (CCA) and the external carotid artery (ECA) were carefully isolated and ligated tightly with a suture. After the ECA branch was cauterized, the internal carotid artery (ICA) was isolated and the pterygopalatine artery was coagulated. To initiate the ischemia, a silicone-coated 4-0 nylon monofilament suture (Doccol Co.) was inserted into the CCA and advanced into the ICA to the origin of the MCA in rats. In the mouse model, a 6-0 nylon monofilament suture was used. The filament was left in place for permanent ischemia, and for the transient model, re-perfusion was conducted by removal of the monofilament at 3 hours after occlusion. Free carnosine or polymersomes in saline were administered intravenously through the lateral tail vein at 3 hours after ischemia onset. In models of transient ischemia, reperfusion was initiated after intravenous treatment of carnosine, polymersomes or corresponding vehicles.

### Calculation of infarct volume using TTC staining

At 24 hours after induction of ischemia, animals were euthanized by isoflurane overdose. Brains were rapidly isolated and then cut into 1 mm sections, stained with triphenyltetrazolium chloride (TTC; 2%), and fixed in 4% paraformaldehyde. Each brain section was scanned to a digital image, and analyzed using the NIH ImageJ software. The infarct volume for each section was calculated and correction for edema was performed by the measurement of the infarcted and control hemisphere.

### Assessment of neurological function

Neurological functional deficit was evaluated by functional tests at 24 hours after MCAo prior to brain isolation. Neurological function was scored from 0 to 18 (normal score, 0; maximal deficit score, 18) by neurological severity scores (NSS). The 18-point-based scale includes the following five tests: spontaneous activity symmetry of movements (maximum 3 points), symmetry of hindlimbs/forelimbs (maximum 3 points), beam balance tests (maximum 6 points), sensory tests (maximum 2 points) and reflexes and abnormal movements (maximum 4 points), as previously described^33^.

### *In vivo* imaging of polymersomes

*In vivo* fluorescence imaging was conducted in BALB/c nude mice (20–25 g). Mice were maintained under isoflurane anesthesia during the fluorescence detection. Polymersomes in a saline suspension was injected intravenously via tail vein, and fluorescence signal (λex: 520 nm, λem: 570 nm) from the whole mouse was monitored in IVIS Lumina XR (Xenogen Corporation Caliper; Waltham, MA, USA) at each time point indicated. To determine the tissue distribution of polymersomes, the fluorescence intensity was measured in each organ. At 24 hours after injection of polymersomes, major organs such as liver, lung, spleen, heart, kidney, and brain were excised and washed with saline before obtaining fluorescent images.

### Sample size estimates

The number of animals to be used per group was determined using a series of power calculations using commercially available software (Janet D. Elashoff, nQuery Advisor, Los Angeles, CA).

### Statistical analysis

All experimental values were expressed as mean and standard deviation (SD). Infarct volumes and neurological scores in graphs were presented using boxplots. Statistical significance between groups was determined by one-way ANOVA with post hoc LSD test^34^. In all analyses, a p < 0.05 was considered statistically significant.
